# The Relationship between Body Dissatisfaction and Eating Disorder Symptoms in Young Women Aspiring Fashion Models: The Mediating Role of Stress

**DOI:** 10.3390/ejihpe11020043

**Published:** 2021-06-18

**Authors:** Sabrina Castellano, Agostino Rizzotto, Sergio Neri, Walter Currenti, Claudia Savia Guerrera, Concetta Pirrone, Marinella Coco, Donatella Di Corrado

**Affiliations:** 1 Department of Education Sciences, University of Catania, 95124 Catania, Italy; sabrina.castellano@unict.it (S.C.); agostino.rizzotto@unict.it (A.R.); concetta.pirrone@unict.it (C.P.); 2 Department of Medicine, University of Catania, 95123 Catania, Italy; sergio.neri@unict.it; 3 Department of Biomedical and Biotechnological Sciences, University of Catania, 95123 Catania, Italy; walter.currenti@studium.unict.it (W.C.); claudia.guerrera@phd.unict.it (C.S.G.); marinella.coco@unict.it (M.C.); 4 Department of Sport Sciences, University Kore of Enna, 94100 Enna, Italy

**Keywords:** fashion models, eating disorders, anorexia, stress

## Abstract

It is widely recognized that body dissatisfaction is an important public health concern. In the past, being a fashion model was almost synonymous with anorexia/bulimia, and even today, there are cases of eating disorders in young women whose ambition is to become a top model. Moreover, stress can play a substantial role within ill health via related behaviors such as smoking, substance abuse, and inappropriate eating. In our study, we examined 112 aspiring fashion models aged between 15 and 24 years (M = 19.5, SD = 2.08) from 32 different countries of the world during an international contest, and 100 students (control group), aged between 16 and 22 years (M = 18.6, SD = 1.39). The purpose of this cross-sectional study was to examine whether stress mediated the relationship between body dissatisfaction and eating disorders. The study included the administration of stress and self-efficacy and the locus of control dimensions, body (image) dissatisfaction, and eating attitude disorder. Results indicated higher scores on body dissatisfaction, stress level, and eating attitudes disorder among the group of fashion models compared to the control. Mediational analyses showed that body dissatisfaction was partially mediated by stress level on eating disorders. Especially in the aspiring fashion models, there are often many possibilities that competitive stress causes candidates to exacerbate attempts to maintain their body weight below normal weight/height parameters. These results indicated that appropriate intervention for the management of stress level could possibly defend against the negative impact of body dissatisfaction on eating disorder symptoms. The presence of skilled health workers in the field of nutrition and psychology can be extremely important in the field of fashion to maintain an adequate quality of life.

## 1. Introduction

Eating disorder [1,2,3] is the medical term used to describe an illness that is characterized by irregular eating habits and severe distress or concern about body weight or shape. It can develop during any stage in life but typically appears during the teen years or young adulthood [4].

It is widely recognized that negative body image is an important public health concern for societies globally because of its reliable association with symptoms of disordered eating and poorer psychological well-being. In the field of eating disorders [5,6,7,8], elevated-risk groups might contain persons whose behavioral patterns feature as definite backgrounds of the future increase of an eating disorder, such as extreme dieting, professional pressure to be skinny, and undue exercise. On these estates, athletes [9,10,11,12,13] and ballet dancers [14,15,16] were widely examined to recognize the main associates of the risk of eating disorders. In the past, and still today, professional fashion models are “usually considered” as beautiful women seeking success by using their charm and their own body. People often think of these women as being stressed-out individuals willing to sacrifice both their physical and intellectual health for their career. Some studies [17,18,19], demonstrated that fashion models have a positive body image and generally do not report more dysfunctional eating behaviors than controls. However, fashion models were on average slightly underweight with significantly lower BMI than controls. Groesz [20] reported that the prevalence of anorexia nervosa and higher prevalence of partial-syndrome eating disorders were significantly higher among professional fashion models compared to controls. Santonastaso [21] and Preti [22] have shown that a high body (image) dissatisfaction and low self-efficacy were associated to health-related complications, such as stressor indicators and elevated risk of clinical eating disorders, in a sample of fashion models compared to their peers as a consequence of the persistent pressure to preserve a thin silhouette. However, further studies [23,24] have failed to find significant differences between professional fashion models and controls on such dimensions as eating-disordered behavior. Even though different studies have investigated the relationship between body dissatisfaction and eating disorder symptoms, coming to different conclusions, the relationship is still far from being clear, particularly regarding a probable factor that could potentially mediate it.

Therefore, this study aimed to examine the relationship between body dissatisfaction and eating disorder symptoms and the mediating role of stress level in a sample of aspiring fashion models. More specifically, we hypothesized the following:

**Hypothesis** **1.** *There are significant relationships among stress level, body dissatisfaction, and eating disorders in fashion models compared to controls.*

**Hypothesis** **2.** *Stress mediates the relationship between body dissatisfaction and eating disorders.*

## 2. Materials and Methods

### 2.1. Participants

In the present study, 112 fashion models from 32 different countries, aged between 15 and 24 years (M = 19.5, SD = 2.08), participated in the study. We compared this group with a voluntary, well-matched control group (same age and cultural background) that composed of 100 Italian young women, aged between 16 and 22 years (M = 18.6, SD = 1.39), recruited in some high schools (n = 50) and in some faculties (n = 50) of the university. 

Both the models, students, and their agents or parents (in the case of minors) were advised that the investigation concerned eating disorders and attitude toward the body. All subjects who agreed to participate in the study, after written informed consent had been obtained both from them and their agents or parents, were requested to complete a set of questionnaires, to attend a specific interview about their eating habits both in everyday life and in relation to periods that preceded stressful situations (exams, parties, dances for the students; before, during, and after participation in fashion events for young women). The anthropometric measurements (weight, height, and BMI) of all participants were taken over by the medical team. This study was carried out in accordance with the recommendations of the Ethical Code of the University of Palermo and of the Code of Ethics approved by the General Assembly of the Italian Association of Psychology held on 27 March 2015.

### 2.2. Procedure

During the final of “The Look of the Year” 2018 (an international competition which elects the Model of the Year through the selection of young women whose ambition is to become a top model as life work), a group of researchers decided to investigate all aspiring fashion models. In the present study, we applied the Diagnostic and Statistical Manual of Mental Disorders (5th ed.; DSM–5) [2] for the identification of full syndrome anorexia and bulimia nervosa. Partial-syndrome anorexia nervosa was detected in cases of underweight according to the subsequent symptoms: amenorrhea, influence of body shape, or fear of getting fat; partial-syndrome bulimia nervosa was diagnosed in cases of binge eating (about eight times per month in the earlier three months) and self-esteem concerning the influence of body shape, or recurrent purging behavior (about eight times per month in the earlier three months). Before the interview, all participants received the set of self-report inventories to evaluate both abnormal eating attitudes and behaviors and related psychological symptoms [22]. Measurements were conducted individually or in small groups of four or five participants (approximately 15 min) in a quiet room, under the supervision of two researchers. Body weight and height were collected through a portable digital scale and stadiometer in another private room. All participants performed a battery of tests and confidentiality of the answers was assured. Fashion models were tested in a secluded location in the place of competition (The Palace Hotel). The participants of the control group were from different classes and were tested in a separated location in either the university or the school at the end of lecture. For the younger participants, a consent form was signed by their parents.

### 2.3. Measures

#### 2.3.1. Body Satisfaction

To measure the body satisfaction, we used [25] the Eating Disorder Inventory-Body Dissatisfaction subscale (EDI-BD) formed by 10 items that assess discontentment with the overall shape and size of those regions of the body of extraordinary concern to those with eating disorders (i.e., stomach, hips, thighs, buttocks), using a 6-point Likert-type scale (from 1 = never to 6 = always), calculating the final average of the scores (a high average reflects high body dissatisfaction). One item on the BD scale measures the feeling of bloating after eating a normal meal, a common feature of those who are dissatisfied with their body weight. The internal consistency of the item scores was satisfactory, showing an α value >0.80.

#### 2.3.2. Bulimic Investigatory Test, Edinburgh (BITE)

The Bulimic Investigatory Test, Edinburgh (BITE) is a measure [26] of bulimic pathology consisting of 33 items related to the presence/absence of behaviors and attitude associated with bulimic disorders. The items consist of (1) a Symptom subscale that assesses the degree of symptoms present and (2) a Severity subscale that indicates the severity of binge eating based on frequency. Answers to the questions are given scores, with a higher score indicating a higher severity or more frequent occurrence. Items are answered in a binary yes or no format. Internal consistency is satisfactory.

#### 2.3.3. Measuring Psychological Stress (PSM-9)

The Measuring Psychological Stress (PSM-9) is a 9-item self-administrated questionnaire [27] directly measuring the state of perceived stress. The items are scored on an 8-point scale (1 = not at all, 8 = extremely). Items include: I feel calm, and I feel full of energy and keen. The internal consistency was satisfactory, showing a Cronbach α coefficient of 0.92 and 0.93.

#### 2.3.4. Eating Attitudes Test (EAT-26)

The Eating Attitudes Test (EAT-26) is a 26-item standardized test [3] that measures symptoms and concerns that are characteristic of eating disorders. The EAT-26 is rated on a six-point scale based on how often the individual engages in specific behaviors (6 = always, 1 = never). Completing the EAT-26 produces a “referral index” based on the following criteria: (1) the total score based on the answers to the EAT-26 questions; (2) the answers to the behavioral questions related to eating symptoms and weight loss; and (3) the individual’s body mass index (BMI) calculated by their height and weight. A cut-off ≥20 indicates the presence of clinically significant eating pathology. The sensitivity and specificity of the EAT-26 in the young Italian people were valued, correspondingly, at 0.50 and 0.95.

#### 2.3.5. Body Mass Index

Body Mass Index (BMI) was computed from self-reported values of weight and height using the standard formula of weight (kg)/height^2^ (m).

### 2.4. Statistical Analysis

Statistical evaluation was carried out using SPSS 23.0 (SPSS Inc, Chicago, Illinois, USA). The level of significance for all analyses was set at α = 0.05. Means and standard deviations are reported for sample descriptions. Student’s t-test was used to detect the means’ differences between the models and the controls. Since most of the variables are not normally distributed, Spearman’s rho correlations are described for the associations between the selected variables. The mediation analysis was performed using Hayes’ [28] PROCESS macro for SPSS (version 3.1). Direct and indirect effects are quantified using ordinary least square (OLS) regression-based path analysis. Inference about the indirect effect is determined by bootstrapping, reporting a 95% bootstrap confidence interval (CI) of 95% [29]. The number of bootstrap samples was set at 5000.

## 3. Results

Twelve aspiring fashion models declined to participate to the study. The Levene’s test did not reveal any significant differences among the two groups in terms of age (F(1, 198) = 20.51, *p* = 0.06, partial η^2^ = 0.57, and BMI (F(1, 198) = 0.24, *p* = 0.07, partial η^2^ = −0.06).

### 3.1. Group Differences and Correlations

Table 1 has reported the anthropometric characteristics of the participants. According to the World Health Organization, a BMI of 18.5–24.9 is considered normal; a BMI less than 18.5 indicates underweight.

Table 2 showed the comparison between models and controls conducted through the Student’s *t*-test (*p* < 0.05).

As seen, for aspiring fashion models, a Student’s *t*-test showed significantly higher scores on body dissatisfaction (*p* = 0.001), stress level (*p* = 0.001), and eating attitudes disorder (*p* = 0.001) than the controls.

The relationships between the variables of the study were analysed using Spearman’s rho correlation coefficient (Table 3).

As can be observed, scores for stress level and eating attitudes disorders were significantly and negatively associated with scores on body dissatisfaction. On the other hand, scores for stress level and eating attitudes disorders were significantly and positively related to attitude associated bulimic disorders. Finally, we found very strong and positive relationships between eating attitudes disorders and body dissatisfaction. The skewness and kurtosis of the distribution of the total scores of the dimensions of the study were calculated: The skewness values were between 0.38 and 0.88; the kurtosis values were between −1.08 and 0.08.

### 3.2. Mediation Analysis

Based on the correlation results, a mediation analysis was executed by entering the score of stress as a mediator in the relationship between body dissatisfaction and eating attitudes disorders. The mediation results are presented in Table 4, which contains the standardized β, indicating the intensity of the effect, and the 95% CIs, indicating the significance of the effect with a 5% probability of error (CIs that do not contain 0 are significant). Mediational analyses showed that body dissatisfaction had both direct and indirect effects through stress level on eating disorders. Thus, the relationship was partially mediated by stress (confirming our hypothesis).

## 4. Discussion

The present study aimed to examine the relationship between body dissatisfaction and eating disorder symptoms, and the mediating role of stress level in a sample of aspiring fashion models. Data analysis partially confirmed the research hypotheses.

More specifically, we observed that stress level partially mediated the relationship between body dissatisfaction and eating attitudes disorders.

There are many health consequences of being underweight. Leptin decreases as body weight falls. Without adequate levels of leptin the sequence of hormonal events that controls ovulation and implantation becomes disrupted. Menstruation becomes irregular or absent and fertility is diminished; poor nutrition stunts bone development (in the growth phase) and reduces bone turnover and repair, leading to osteoporosis (the impact on bones in eating disorders is a clear exemplar of these effects). Even in the adolescent population [1,2,3,4,5,30], a negative body image perception and poor self-esteem may result in health-related effects, such as stressor symptoms and greater risk of clinical eating disorders. Previous studies [31,32] indicated that the prevalence of body image dissatisfaction in developed countries ranges between 35% and 81% among female adolescents and between 16% and 55% among male adolescents. In relation to the fashion models, in addition to the biological factors described above, even social factors contribute to the unhealthy lifestyle common among those pursuing a modelling career. Constant exposure to media images depicting thin women reduces body-related satisfaction, particularly in young aspiring models. Ralph-Nearman and colleagues [33] found an earlier formal diagnosis of anorexia nervosa in a sample of fashion models compared to their peers. Particularly, they reported an indicator of a higher risk of a partial eating disorder disease. According to another study [34], young women susceptible to eating disorders and who are highly competitive tended to be more hostile and stressed than their fellows. Chen and coworkers [35] examined the direct pathway in the relationship between body dissatisfaction and eating disorder symptoms and the possible mediation pathways between the two via regulatory emotional self-efficacy and depression symptoms in 654 adolescents. The results showed that, in addition to the direct pathway between body dissatisfaction and eating disturbances, regulatory emotional self-efficacy and depression symptoms partially mediated the relationship between these two constructs. Other researchers have explored the roles of social appearance anxiety and emotional intelligence in the relationship between body esteem and eating disorder risk among adolescents [36]; the roles of self-esteem and negative affect in the relationship between body dissatisfaction and disordered eating in 806 adolescents [37]; and the roles of relevant psychological (cognitive and emotional) predictors of binge eating behaviors in 120 adolescents [38].

A far as we know, no studies have evaluated the relationship between body dissatisfaction and eating disorder symptoms and the mediating role of stress level in aspiring fashion models compared with a population of non-models of the same age. The results of our study indicated a higher prevalence of dysfunctional eating behaviors, body dissatisfaction, and stress level in fashion models compared to controls. Moreover, fashion models had a slightly lower than average BMI (below 18) than the controls, indicating the cut-off for under-nutrition. In addition, as suggested in our results, we believe that the difference in professionalism between models and aspiring models can be very significant. In fact, the second category can be much more exposed than the first ones to the risks that may arise from poor control of the diet and exasperated weight lookup because of the younger age, lesser experience, uncertainty of their future, and possible competitive exasperation that can diminish their resilience to stress. In a beauty contest, the competitive stress can cause candidates to exacerbate attempts to maintain their body weight below normal weight/height parameters. This attitude can always “be lurking” and sometimes can bring on conditions of food disorders. For this reason, the presence of skilled health workers in the field of nutrition and psychology can be extremely important in the field of fashion in relation to the training of girl models where competitiveness and stress are unavoidable conditions. Any wrong eating habits or psychological conflict linked to the exasperation of the competitions can be corrected by the proximity of skilled health workers; they can, on the one hand, help to change any unhealthy diet and optimize the daily nutritional intake in compliance with the requirements related to that specific profession. On the other hand, they can teach and/or improve resilience to the inevitable stress caused by competition targeted to the choice of a future model.

## 5. Limitations

Of course, the present findings should be interpreted in the light of some limitations of our work. First, the group of models examined was certainly representative of most of the world’s countries, but no equal number of models were expected for each nation and this could determine some bias related to the dietary habits of each different nation. Second, as with any study using self-report measures completely, its results may be vulnerable to selective or erroneous reporting. Moreover, we used a subscale (Body Dissatisfaction) of the same scale (i.e., EDI). Even though the subscales are indicated as distinct constructs, measures designed as subscales reproducing a common higher order construct (i.e., eating disorder symptomatology) may contain overlapping variance that would not be present in separate scales assessing the primary constructs of interest. Another limitation of the present design was that we focused on key indices of body image and appearance-related concerns, but these may have concealed other differences between models and our control group.

## 6. Conclusions

In conclusion, research is required to interpret the complex dimensions of body dissatisfaction as well as its implications. Particularly, it is evident that there are differences between “achievable” and “unachievable” body sizes. Although discrepancy between one’s current body size and either of these constitutes body dissatisfaction, experience of each carries unique consequences for practitioners. Finally, we believe that interactive lessons on the psychological risks of an unhealthy diet and an ongoing psychological counseling before, during and after the competition can be extremely helpful to avoid in these young women the risks of eating behavior disorder. The fashion and beauty industry can play a key role in preventing the development of unhealthy lifestyles in young people.

## Figures and Tables

**Table 1:** Participants’ anthropometric characteristics. Mean ± *s* (range).

Groups	Age	BMI	Weight (kg)	Height (cm)
Models	19.5 ± 2.08 (15–24)	18.4 ± 1.05 (17.2–21.3)	57 ± 4.21 (49.1–66.0)	176 ± 4.06 (170–183)
Controls	18.6 ± 1.39 (16–22)	19.6 ± 1.28 (18.2–23.0)	62 ± 2.54 (55.0–68.0)	170 ± 5.33 (158–180)

**Table 2:** Group differences in the dimensions of the study (Student’s *t*-test, *p* < 0.05).

	Models		Controls				
	M	SD	M	SD	*t*	*p*	Cohen’s d
EDI-BD	29.46	6.21	14.70	3.67	−20.44	0.001	−2.89
BITE	12.75	2.79	12.93	3.66	−0.39	0.696	−0.05
PSM-9	33.75	10.26	20.36	5.83	11.34	0.001	1.60
EAT-26	11.73	4.95	7.96	4.1	5.86	0.001	0.82

Notes: EDI-BD is Eating Disorder Inventory-Body Dissatisfaction subscale; BITE is Bulimic. Investigatory Test; PSM-9 is Measuring Psychological Stress; EAT-26 is Eating Attitudes Test.

**Table 3:** Correlation coefficients (Spearman’s rho).

Variable	1	2	3	4
1. EDI-BD	1			
2. BITE	−0.086	1		
3. PSM-9	−0.545 ***	0.261 ***	1	
4. EAT-26	−0.356 ***	0.340 ***	0.451 ***	1

Note. EDI-BD is Eating Disorder Inventory-Body Dissatisfaction subscale; BITE is Bulimic Investigatory Test; PSM-9 is Measuring Psychological Stress; EAT-26 is Eating Attitudes Test. *p* < 0.05, *p* < 0.01, *** *p* < 0.001.

**Table 4:** Effects of body dissatisfaction on eating attitudes disorders through stress (standardized β).

Paths	Indirect Effect				Direct Effect			Total Effect	
	β	C.I. 95%	*p*	β	C.I. 95%	*p*	β	C.I. 95%	*p*
Body dissatisfaction–Stress-Eating disorders	−0.022	−0.034~ #x2212;0.012	0.001	−0.017	−0.036~ #x2212;0.009	0.03	−0.040	−0.054~−0.026	0.001

## References

[1] Rosenvinge J.H., Pettersen G. (2014). Epidemiology of eating disorders part II: An update with a special reference to the DSM-5. Adv. Eat. Disord..

[2] American Psychiatric Association (2006). Diagnostic and Statistical Manual of Mental Disorders.

[3] Garner D.M., Olmsted M.P., Bohr Y., Garfinkel P.E. (1982). The Eating Attitudes Test: Psychometric features and clinical correlates. Psychol. Med..

[4] Bibiloni M.D.M., Pich J., Pons A., Tur J.A. (2013). Body image and eating patterns among adolescents. BMC Public Health.

[5] Cooper Z., Fairburn C. (1987). The eating disorder examination: A semi-structured interview for the assessment of the specific psychopathology of eating disorders. Int. J. Eat Disord..

[6] Striegel-Moore R.H., Bulik C.M. (2007). Risk factors for eating disorders. Am. Psychol..

[7] Hoek H.W., Fairburn C.G., Brownell K.D. (2002). Distribution of eating disorders. Eating Disorders and Obesity: A Comprehensive Handbook.

[8] Schaumberg K., Welch E., Breithaupt L., Hübel C., Baker J.H., Munn-Chernoff M.A., Yilmaz Z., Ehrlich S., Mustelin L., Ghaderi A. (2017). The Science Behind the Academy for Eating Disorders’ Nine Truths About Eating Disorders. Eur. Eat. Disord. Rev..

[9] Kirchhoff J., Filippi A. (2015). Comparison of oral health behavior among dental students, students of other disciplines, and fashion models in Switzerland. Swiss Dent. J..

[10] Röthlin P., Birrer D., Horvath S., Holtforth M.G. (2016). Psychological skills training and a mindfulness-based intervention to enhance functional athletic performance: Design of a randomized controlled trial using ambulatory assessment. BMC Psychol..

[11] Smolak P., Murnen S.K., Ruble A.E., Holtforth M.G. (2000). Female athletes and eating problems: A meta-analysis. Int. J. Eat. Disord..

[12] De Oliveira G.L., de Oliveira T.A.P., de Pinho Gonçalves P.S., Valentim Silva J.R., Roquetti Fernandes P., Fernandes Filho J. (2017). Body Image and Eating Disorders in Female Athletes of Different Sports. J. Exerc. Physiol..

[13] De Bruin A., Oudejans R.R.D. (2018). Athletes’ Body Talk: The Role of Contextual Body Image in Eating Disorders as Seen Through the Eyes of Elite Women Athletes. J. Clin. Sport Psychol..

[14] Cardoso A.A., Reis N.M., Moratelli J., Borgatto A., Resende R., Guidarini F.C.D.S., Guimarães A.C.D.A. (2021). Body Image Dissatisfaction, Eating Disorders, and Associated Factors in Brazilian Professional Ballroom Dancers. J. Dance Med. Sci..

[15] Cardoso A.A., Reis N.M., Marinho A.P., Boing L., Guimarães A.C.D.A. (2017). Study of Body Image in Professional Dancers: A Systematic Review. Rev. Bras. Med. Esporte.

[16] Walter O., Yanko S. (2018). New observations on the influence of dance on body image and development of eating disorders. Res. Dance Educ..

[17] Swami V., Szmigielska E. (2013). Body image concerns in professional fashion models: Are they really an at-risk group?. Psychiatry Res..

[18] De Freitas C., Jordan H., Hughes E.K. (2018). Body image diversity in the media: A content analysis of women’s fashion magazines. Health Promot. J. Aust..

[19] Ahern A.L., Bennett K.M., Hetherington M.M. (2008). Internalization of the Ultra-Thin Ideal: Positive Implicit Associations with Underweight Fashion Models are Associated with Drive for Thinness in Young Women. Eat. Disord..

[20] Groesz L.M., Levine M.P., Murnen S.K. (2002). The effect of experimental presentation of thin media images on body satisfaction: A meta-analytic review. Int. J. Eat. Disord..

[21] Santonastaso P., Mondini S., Favaro A. (2002). Are Fashion Models a Group at Risk for Eating Disorders and Substance Abuse?. Psychother. Psychosom..

[22] Preti A., Usai A., Miotto P., Petretto D.R., Masala C. (2008). Eating disorders among professional fashion models. Psychiatry Res..

[23] Zancu S.A., Enea V. (2017). Eating disorders among fashion models: A systematic review of the literature. Eat. Weight Disord. Stud. Anorex. Bulim. Obes..

[24] Treasure J.L., Wack E.R., Roberts M.E. (2008). Models as a high-risk group: The health implications of a size zero culture. Br. J. Psychiatry.

[25] Clausen L., Rosenvinge J.H., Friborg O., Rokkedal K. (2010). Validating the Eating Disorder Inventory-3 (EDI-3): A Comparison Between 561 Female Eating Disorders Patients and 878 Females from the General Population. J. Psychopathol. Behav. Assess..

[26] Orlandi E., Mannucci E., Cuzzolaro M., SISDCA-Study Group on Psychometrics (2005). Bulimic Investigatory Test, Edinburgh (BITE). A validation study of the Italian version. Eat. Weight Disord. Stud. Anorex. Bulim. Obes..

[27] Lemyre L., Lalande-Markon M.-P., McLaughlin Research Chair (2009). Psychological stress measure (PSM-9): Integration of an evidence-based approach to assessment, monitoring, and evaluation of stress in physical therapy practice. Physiother. Theory Pr..

[28] Hayes A.F. (2013). Introduction to Mediation, Moderation, and Conditional Process Analysis: A Regression-Based Approach.

[29] Preacher K.J., Kelley K. (2011). Effect size measures for mediation models: Quantitative strategies for communicating indirect effects. Psychol. Methods.

[30] Lawler M., Nixon E. (2011). Body dissatisfaction among adolescent boys and girls: The effects of body mass, peer appearance culture and internalization of appearance ideas. J. Youth Adolesc..

[31] Duchin O., Marín C., Mora-Plazas M., de Leon C.M., Lee J.M., Baylin A., Villamor E. (2014). A prospective study of body image dissatisfaction and BMI change in school-age children. Public Health Nutr..

[32] Radwan H., Hasan H.A., Ismat H., Hakim H., Khalid H., Al-Fityani L., Mohammed R., Ayman A. (2019). Body Mass Index Perception, Body Image Dissatisfaction and Their Relations with Weight-Related Behaviors among University Students. Int. J. Environ. Res. Public Health.

[33] Ralph-Nearman C., Yeh H.-W., Khalsa S.S., Feusner J.D., Filik R. (2020). What is the relationship between body mass index and eating disorder symptomatology in professional female fashion models?. Psychiatry Res..

[34] Miotto P., de Coppi M., Frezza M., Petretto D.R., Masala C., Preti A. (2003). Eating disorders and aggressiveness among adolescents. Acta Psychiatr. Scand..

[35] Chen G., He J., Zhang B., Fan X. (2021). Revisiting the relationship between body dissatisfaction and eating disorder symptoms in Chinese adolescents: The mediating roles of regulatory emotional self-efficacy and depression symptoms. Eat. Weight Disord. Stud. Anorex. Bulim. Obes..

[36] Li Y. (2019). Linking body esteem to eating disorders among adolescents: A moderated mediation model. J. Health Psychol..

[37] Cruz-Sáez S., Pascual A., Wlodarczyk A., Echeburúa E. (2018). The effect of body dissatisfaction on disordered eating: The mediating role of self-esteem and negative affect in male and female adolescents. J. Health Psychol..

[38] Di Nardo M., Marchetti D., Fulcheri M., Verrocchio M.C. (2020). Understanding the link between interoceptive deficits and binge eating symptoms among adolescents: A serial mediation analysis. Mediterr. J. Clin. Psychol..

